# Inflammation and Liver Cell Death in Patients with Hepatitis C Viral Infection

**DOI:** 10.3390/cimb43030139

**Published:** 2021-11-16

**Authors:** Manuela G. Neuman, Lawrence B. Cohen

**Affiliations:** 1 In Vitro Drug Safety and Biotechnology, The Department of Pharmacology and Toxicology, Temerity Faculty of Medicine, University of Toronto, Banting Institute, 100 College St., Toronto, ON M5G 1L5, Canada; 2 Sunnybrook Health Sciences Centre, Division of Gastroenterology, Department of Medicine, Temerity Faculty of Medicine, 2075 Bayview Ave., Toronto, ON M4N 3M5, Canada; lawrence.cohen@sunnybrook.ca

**Keywords:** tumor necrosis factor alpha, apoptosome, inflammasome, cytokines, chemokines, fibrosis, viral hepatitis C, electron microscopy

## Abstract

Hepatitis C virus (HCV)-induced liver disease contributes to chronic hepatitis. The immune factors identified in HCV include changes in the innate and adaptive immune system. The inflammatory mediators, known as “inflammasome”, are a consequence of the metabolic products of cells and commensal or pathogenic bacteria and viruses. The only effective strategy to prevent disease progression is eradication of the viral infection. Immune cells play a pivotal role during liver inflammation, triggering fibrogenesis. The present paper discusses the potential role of markers in cell death and the inflammatory cascade leading to the severity of liver damage. We aim to present the clinical parameters and laboratory data in a cohort of 88 HCV-infected non-cirrhotic and 25 HCV cirrhotic patients, to determine the characteristic light microscopic (LM) and transmission electron microscopic (TEM) changes in their liver biopsies and to present the link between the severity of liver damage and the serum levels of cytokines and caspases. A matched HCV non-infected cohort was used for the comparison of serum inflammatory markers. We compared the inflammation in HCV individuals with a control group of 280 healthy individuals. We correlated the changes in inflammatory markers in different stages of the disease and the histology. We concluded that the serum levels of cytokine, chemokine, and cleaved caspase markers reveal the inflammatory status in HCV. Based upon the information provided by the changes in biomarkers the clinician can monitor the severity of HCV-induced liver damage. New oral well-tolerated treatment regimens for chronic hepatitis C patients can achieve cure rates of over 90%. Therefore, using the noninvasive biomarkers to monitor the evolution of the liver damage is an effective personalized medicine procedure to establish the severity of liver injury and its repair.

## 1. Introduction

### 1.1. Viral Hepatitis C

Chronic HCV infection is a major cause of liver-related morbidity and mortality. Most patients are diagnosed based on the presence of elevated transaminases concomitantly with the presence of HCV ribonucleic acid (HCV-RNA) [1,2,3,4,5]. HCV infection leads to chronic hepatitis, and progresses to liver cirrhosis and hepatocellular carcinoma [3].

HCV end-stage liver disease has become the leading indication for liver transplantation, accounting for approximately half of the transplants performed in European and North American centers [6]. Factors related to the virus, the host, and the donor are implicated in the outcome [6,7,8]. The link between hepatitis virus, alcohol consumption, and disease severity is demonstrated [9,10,11,12,13,14,15,16,17,18,19,20]. HCV is also connected to an increase in cytokine and chemokine serum levels [21,22,23]. In HCV sera, there are higher levels of alanine aminotransferase (ALT) than aspartate aminotransferase (AST) [10].

### 1.2. Liver Disease and the Cytokine Storm Syndrome

Liver damage due to bacteria, viruses, and drug misuse leads to the recognition of a diverse range of stress signals by inflammasomes. The result of this is the activation of caspase-1, which subsequently induces the secretion of potent pro-inflammatory cytokines and a form of programmed cell death via apoptosis or pyroptosis. Inflammasome-mediated processes regulate both metabolic processes and mucosal immune responses [13,14,15,16]. As a consequence, it is important to monitor cytokine production and signaling pathways during liver inflammation and repair. An additional consideration should be given to the fact that some of the individuals need several therapeutics for their conditions [15,16,17,18,19,20,21]. The use of antibiotics can change their gut microbiome [22]. Many therapeutics have the potential to interact adversely with alcohol, leading to cytokine release into the blood [15,22,23,24]. The humoral immune response functions through cytokines, which regulate macrophages, natural killer cells (NkT) and antiviral cellular proteins. Cytokines produced in the liver are an integral part of the host’s defense to invasion by HCV. 

Persistent infection upsets the balance between immunostimulatory and inhibitory cytokines, leading to cell necrosis and fibrosis of the liver [18,19]. Tumor necrosis factor (TNF-α) and interleukin (IL)-6 contribute to inflammation [20,21]. In addition, interlekin-17 (IL-17) signals for the inflammation of Kupffer cells and hepatic stellate cells. This leads to the exacerbation of fibrosis [22]. Moreover, the changes in the levels of metaloproteinases enhance fibrosis of the liver [23]. The chemokine CCL5/RANTES (regulated upon activation normal T cell expressed and presumably secreted) contributes to the progression of injury during chronic liver disease, and leads to hepatocellular carcinoma (HCC) [24].

Hepatocytes express and secrete chemokines and damage-associated molecular patterns (DAMPS). HCV-infected cells produce cytokine storm syndrome, characterized by the release of pro-inflammatory cytokines [17,18,19,20].

The inflammasome is composed of procaspase (pro-CASP-1). The PYCARD and NOD-like receptor pyrin domain-containing 3 (NLRP) mediate pro-CASP1 activation. Active CASP1 induces pro-IL-1β and pro-IL-18. The presence of the damage-associated molecular pattern (DAMP)-mediated activation of pro-IL-1β in the steatotic liver is pro-inflammatory. In addition, IL-1β activates matrix metalloprotease (MMP9). MMP is responsible for matrix remodeling and hepatic stellate cell (HSC) activation. An additional pathway of the inflammasome is via caspase (CASP4/11-GSDMD); this pathway is responsible for programmed lytic cell death, “pyroptosis”. Important inducers include viral and bacterial products such as pathogen-associated molecular patterns (PAMPs) and virulence factors. PAMPs elicit inflammation through the recognition of innate pattern recognition receptors (PRRs), whereas virulence factors trigger inflammation via functional feature recognition. PAMPs and DAMPs include products of extracellular matrix (ECM) breakdown [25].

Increasing evidence suggests an important role for hepatocyte apoptosis in the progression of liver damage [26,27]. Several other forms of cell death have been described, including necrosis, necroptosis, autophagic cell death, and others [28]. Both apoptosis and necrosis are responsible for the progression of liver fibrosis [29]. Caspases are activated early in the process. They cleave various substrates, including keratin 18 (K18) [30,31]; K18 is a cytoskeletal protein [32]. Together with K8, K18 represents the major intermediate filament protein in liver cells [33]. K18 is important for the maintenance of hepatocyte integrity since deficiency of intact K18 results in liver damage and dysfunction [34]. The cleavage of K18 exposes the following two epitopes: M30, a marker of apoptosis (caspase 3-cleaved K18) and M65 released from dying cells, regardless of if the process is apoptosis, necrosis, or pyroptosis [30]. The pyroptosis pathway links cell death to local inflammation. This pathway is activated by intracellular lipopolysacharides (LPS).

**Figure 1:**
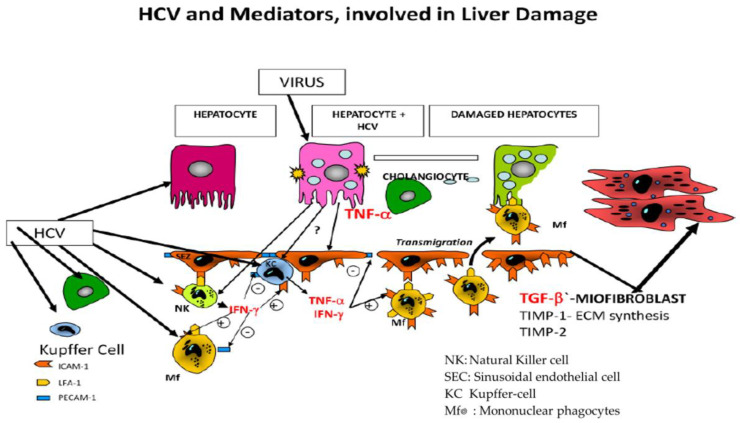
Hepatocytes, sinusoidal epithelial cells (SEC), macrophages [(Kupffer cells (KC)] and stellate cells are the main cells in the liver. Transformations of stellate cells into myofibroblasts is influenced by the following proinflammatory cytokines: interferon alpha and gamma (IFN-α; IFNγ). TNF α, and vascular endothelial growth factor (VEGF). Tissue inhibitors of metalloproteinases (TIMP 1 and 2) and transforming growth factor (TGF−β) contribute to modulating liver fibrosis.

Figure 1 presents schematic factors that contribute to the transformation of hepatic stellate cells into myofibroblasts in an HCV-infected liver.

To evaluate the inflammasome in HCV we quantified the serum levels of Interferon (IFNγ), interleukin-6 (IL-6), nuclear factor-κB (NFκB), regulated upon activation normal T cell expressed and secreted (RANTES), TNF-α, TGF−β, and VEGF.

## 2. Aims

We aimed: to follow the clinical parameters and laboratory data in a cohort of HCV-infected patients, deciphering the severity of liver damage with inflammatory apoptosis (M30) and necroptosis (M65) in the serum of the patients; to determine the characteristic LM and TEM changes in their liver biopsies and to present the link between the severity of liver damage and the serum levels of cytokines. Presenting these non-invasive biomarkers to clinicians will inform and help them to assure positive outcomes for patients. 

## 3. Materials and Methods

### 3.1. Patient Cohort and Clinical Data

The controls and patients with HCV were Caucasians, Blacks and Asians with no viral hepatitis B (HBV) or human immunodeficiency (HIV) viral infection. Besides different degrees of liver disease severity, HCV patients did not present comorbid conditions. All the patients were offered the standard of care. They agreed to have their clinical and laboratory data collected and analyzed. In addition, they consented to publication of the data.

We compared the inflammation in HCV individuals with a control group of 280 healthy individuals who were 30 ± 12 years of age (45% male and 55% female). The control group were individuals who voluntarily gave blood samples as controls for their relatives who underwent different analyses in our laboratory. All the controls agreed to have the data published. 

The body mass index (BMI) of the control group was 20.0 ± 5.0 kg/m^2^. The biochemical and hematological parameters were in the normal limit, as follows: aspartate amno-transferase-AST (<50 U/L); alanine aminotransferase-ALT (<50 U/L); gamma glutamyl transferase-GGT (<60 U/L); alkaline phosphatase-AP (<130 U/L); bilirubin total (<1.2 mg/dL); international normalized ratio-INR (<1.15); creatinine (>13.5 mg/dL); hemoglobin (>15 g/dL); platelets (>150/nL).

HCV-infected patients presented fibrosis F2–F3 on METAVIR score. Clinical evidence of liver decompensation and cirrhosis on biopsy were exclusionary. 

We compared the non-cirrhotic individuals with a group of 25 HCV cirrhotic patients. Other exclusion criteria included HBV or HIV co-infection, alcohol consumption >40 g/week, drug misuse, and injection drug use. Their weights were not below 40 kg or above 125 kg.

In Table 1 we present the AST, ALT, AP, GGT and total bilirubin for the 88 non-cirrhotic and 25 cirrhotic individuals.

The patients presenting HCV infections have genotype 1 HCV (non-cirrhotic: 77% male and 23% female; cirrhotic: 80% male and 20% female). They were part of a group followed up for their routine clinical treatment at the Division of Gastroenterology, Sunnybrook Health Sciences Centre and In Vitro Drug Safety and Biotechnology Laboratory in Toronto, Canada. The routine biochemical and virologic analysis was part of the continued follow-up of the patients. 

The control group represents individuals with normal liver function tests and normal cytokine levels. They were healthy. The controls and patients were non-drinkers.

### 3.2. Special Viral and Cytokine Analysis

Blood samples from all the patients were sent to In Vitro Drug Safety and Biotechnology. The samples were analyzed for the following special virology tests: Epstein–Barr virus (EBV), cytomegalovirus (CMV), human herpes virus 6 and 7 (HHV-6, HHV7) cytokines and chemokines, and apoptosis and necrosis markers [8,9,10,11].

HHV-6 levels were measured as per manufacturer’s instructions using the RealStar^®^ HHV-6 polymerase chain reaction (PCR) kit 1.0 detection, differentiation and quantification reagent system (Altona Diagnostics GmbH, Hamburg, Germany). This test is based on the PCR amplification of specific target sequences and specific target probes, as well as simultaneous detection of PCR amplicons by fluorescent dye-labeled probes. PCR was performed using a Rotor-Gene™ 6000 (Rotor Gene, Corbett Research, Sydney, Australia) using Rotor-Gene 1.7 software (Corbett Research, Sydney, Australia). HHV-6 has been sub-genotyped into genotypes A or B.

The pro- and anti-inflammatory cytokines were quantitatively measured in the sera using available cytokine enzyme-linked immunosorbent assay (ELISA) kits. These are designed to recognize both natural human and recombinant human cytokines. Our laboratory uses special in-house validated controls. The cytokines were as follows: transforming growth factor (TGF)-β (R&D Systems, Inc.; Minneapolis, MN, USA), interleukin (IL)-1, IL-6, IL-8, interferon gamma (IFN-γ), nuclear factor kappa-B (NF-κBp65), regulated upon activation normal T- cell expressed and presumably secreted (RANTES), vascular endothelial growth factor (VEGF), tumor necrosis factor alpha (TNF-α) (pg/mL) (PeproTech Asia, Rehovot, Israel).

Specimens were analyzed in duplicates with 95% sensitivity and 90% specificity. The tests were performed according to manufacturer’s specifications. For cytokine and apoptosis determination, each specimen was analyzed in duplicate with 95% sensitivity and 90% specificity. Our measurement system demonstrates strong correlations across replicates with correlation coefficients >0.99, ensuring reliable detection of differences in cytokine levels between biological samples [18,19,20]. The standards and reference reagents were from the National Institute for Biological Standards and Controls (NIBSC, Herts, UK).

### 3.3. Apoptosis

Liver cell apoptosis is triggered by host conditions and the presence of toxins. To confirm the mechanism, we chose to analyze multiple apopto-necrotic markers. Cytokeratin 18 encodes type I, chain keratin 18. Keratin 18, together with keratin 8, are expressed in single-layer epithelial tissues of the body. 

We measured the cytokeratins in sera using the mitochondrial markers M30 and M65. M30 is specific for apoptosis and M65 combines death processes from both apoptosis and necrosis as described previously [18,19,20]. The M30 Apoptosense^®^ ELISA measures the levels of soluble caspase-cleaved K18 (ccK18) fragments containing the K18Asp396 neo-epitope. The ccK18 level increases during apoptosis and is inhibited by the caspase inhibitor zVAD-fmk M65^®^.

The cytokeratins CK 18 and CK 8 (M30 and M65) were quantified using kits from Bender MedSystems (Vienna, Austria). The correlation coefficient was linear (r = 0.990). These methods are standardized in our laboratory according to the procedures described [17,18,19,20,21]. We used standards and reference reagents available from Bender MedSystems (Vienna, Austria).

The correlation coefficient was linear (r = 0.989) in a concentration range between 2 and 500 pg/mL. The sera with higher concentrations were diluted. 

For a statistical description of the groups, we used mean and standard deviation. Between-group differences were tested for statistical significance using the independent samples *T* test for continuous variables and the chi-square test for binary data. Change in paired data was tested using the paired samples *T* test. Correlation analysis was performed using the Spearman’s rank correlation coefficient. *p* values < 0.05 were considered significant.

### 3.4. Histological Analysis

The biopsies of 20 HCV patients contained liver specimens (biopsy lengths of 16.1 ± 12.5 mm), which were taken for clinical diagnostic purposes. The percutaneous biopsy used the Menghini technique under ultrasound guidance. The tissue was fixed in formalin and embedded in paraffin. The histological analysis was performed in 4 µm sections. The tissue was further dewaxed and stained with hematoxylin and eosin (H&E), using standard procedures. Part of the biopsy was preserved in universal fixative and used for electron microscopy (EM). First, the adequacy of the sample for EM was indicated by the presence of at least 500 hepatocytes in the sample and a minimum length of 2000 microns (2.0 mm) of perisinusoidal space per sample for EM. Quantitation of perisinusoidal cells including stellate cells was made by two independent measurements. First, toluidine blue-stained 1-micron-thin sections were examined under a light microscope. Five unit areas, each containing 100 hepatocytes, were surveyed and the number of fat-storing stellate cells were quantitated. This became the standard stellate cell index, as described by Sztark et al. [35].

The second method of quantification was by direct examination on the screen of the electron microscope and by examination of individual stellate cells on electron microscope-generated photomicrographs. The control liver biopsy tissues were taken from livers of 25 patients with normal histology, who had serum antibodies against HCV, but whose liver biopsies were within normal limits. There were 12 men and 13 women in this group, with ages ranging from 17 to 69 years old. Assessment of perisinusoidal collagenosis was performed by EM examination using the semi-quantitative index established by Blendis et al. [30]. Kupffer cells were also quantitated. This was performed by light microscopic examination of immuno-histochemical-stained slides using antibodies to the CD68 marker for macrophages. Statistical analyses were performed with the Kruskal–Wallis test. A *p* value of less than 0.05 is considered statistically significant in this test.

The EM survey uses the JEOL electron microscope (JEOL Ltd., Tokyo, Japan).

The light microscopy initial survey was performed as a routine that classified each of the biopsies and their degree of inflammation (activity index) or fibrosis. In parallel, toluidine blue-stained thin sections of biopsies were examined under a light microscope at ×1000 magnification (100 oil immersion lens). The number of stellate cells, surface of the lipid droplets in each of the stellate cells, as well as the surface of collagen bundles was quantified by light microscopy. Morphometry was performed using an Olympus microscope equipped with Leco 2005 Image Processing and Analysis System (Leco Instr., Toronto, ON, Canada) with Microsoft program. Cells were considered apoptotic if the pyknotic nuclei could be observed, in addition to the nuclear chromatin fragmentation and cytoplasmic condensation in cells. Normal mitochondrion has a double membrane and regular cristae. The double membrane is lost and the cristae are not observed when the organelle is not functional.

Representative blocks were selected and subjected to ultra-thin sectioning. They were stained with uranyl acetate and lead citrate for transmission electron microscopy (TEM). The investigators overviewed hepatic cords and sinusoids in all biopsies using low-magnification TEM (×750). The following features were studied: bile ducts, bile canaliculi, Kupffer cells, perisinusoidal or stellate cells, and organelles such as mitochondria, rough and smooth endoplasmic reticulum, lysosomes, peroxisomes and plasma membranes.

We also focused on the quantification of apoptotic cells (AC) and apoptotic bodies (AB), as well as the quantity of lipid droplets in stellate cells and the amount of collagen present in the perisinusoidal space. The AC counts were made directly from the viewing screen of the electron microscope. The standard unit of comparison was the number of apoptotic cells per randomly selected unit of 100 hepatocytes. Particular attention was paid to the morphological identification of stellate cells.

### 3.5. Statistical Analysis

For a statistical description of the groups, we used mean and standard deviation. Between-group differences were tested for statistical significance using the independent samples *T* test for continuous variables. Change in paired data was tested using the paired samples *T* test. Correlation analysis was performed using the Spearman’s rank correlation coefficient. *p* values < 0.05 were considered significant.

## 4. Results

### 4.1. Patients Characteristics

#### 4.1.1. Apoptosis and Necrosis and Inflammation

Apoptosis (M-30) and necrosis (M-65) and cytokines in sera of HCV patients and healthy individuals are shown in Table 2.

#### 4.1.2. Correlation of Serum Biomarkers of Apoptosis, Necrosis and Inflammation with Histology

To diagnose the stage of the disease, a biopsy was performed in HCV-infected patients. The biopsy seen by LM presents: tubular inflammation, steatosis and balooning of the hepatocytes. Table 3 presents the correlation between serum and histological markers of liver damage. We used Sperman’s correlation test to analyze the possible link between the marker of inflammation, apoptosis and necrosis measured in serum and the histological features that show liver tissue injury (Table 3).

**Figure 2:**
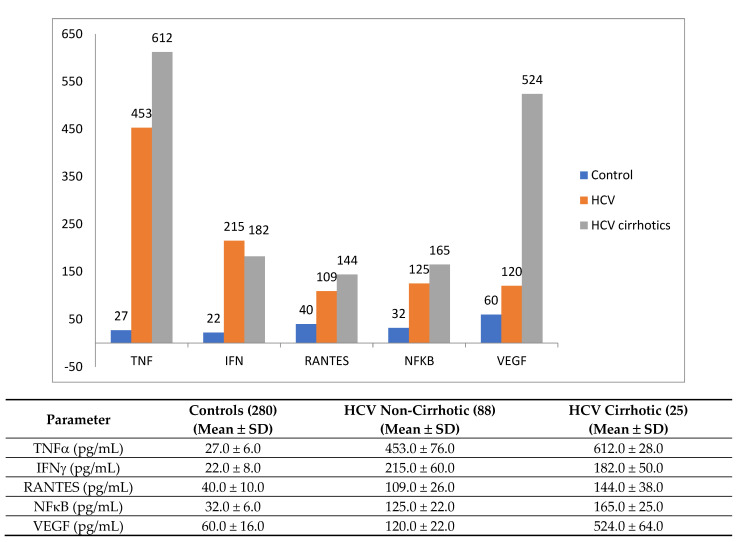
Serum levels of tumor necrosis factor alpha (TNFα), interferon gamma (IFN-γ), regulated upon activation normal T cell expressed and secreted (RANTES) nuclear factor kappa B (NFκB), vascular endothelial growth factor (VEGF) in controls, and in HCV-infected patients. All the parameters are expressed as pg/mL; mL-milliliter; pg-picogram. The levels of TNFα are significantly different between the HCV non-cirrhotic and cirrhotic individuals (*p* = 0.05) and VEGF (*p* = 0.0010).

In Figure 2, we compare the serum levels of TNFα, INFγ, NFκB, and RANTES and VEGF in controls vs. patients.

### 4.2. Histology

The TEM micrographs present several apoptotic hepatocytes and apoptotic bodies (AB). The micrographs show large deposits of lipids in the cell. The micrograph reveals an enlarged smooth endoplasmic reticulum (ER) and numerous mitochondria. While some mitochondria are normal, others have lost their cristae. An HCV antigen can be observed. In addition, the relative number of stellate cells per 100 hepatocytes was more than ten times higher than in the controls. The number of lipid vesicles stored in these stellate cells was 20 times higher than in the controls. Stellate cells in a normal liver only carry between one and four lipid vesicles.

**Figure 3:**
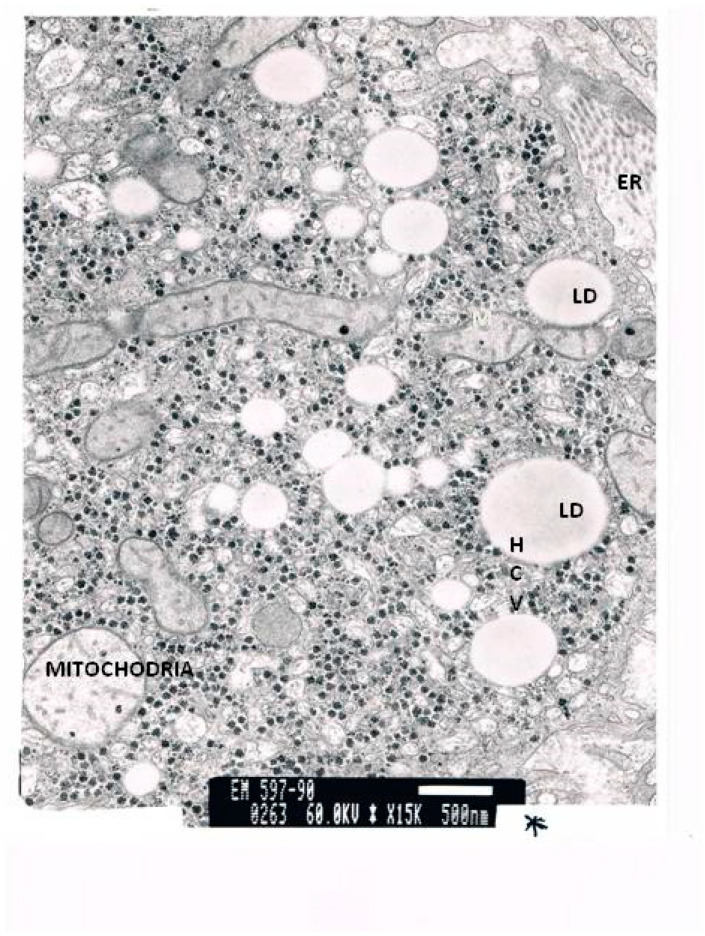
TEM of a liver biopsy. Many giant lipid vesicles and large confluent lipid droplets can be observed. There are many enlarged mitochondria with scattered unregular cristae. HCV antigen can be observed. HCV antibodies are present.

**Figure 4:**
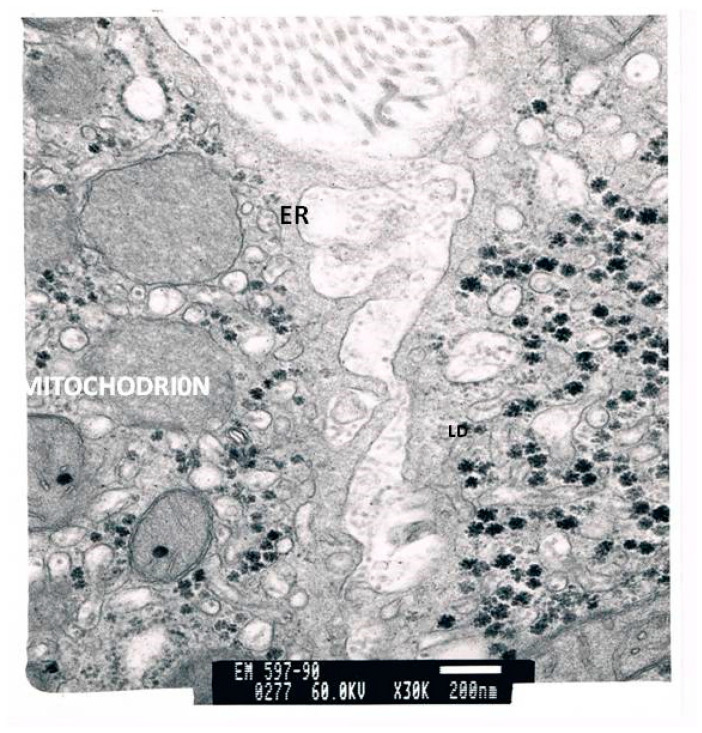
An electron micrograph of a liver biopsy of a patient, which shows hepatocytes containing large lipid droplets, enlarged endoplasmic reticulum, mitochondria with few cristae and HCV antibodies.

Figure 3 and Figure 4 present two different micrographs from a biopsy.

**Figure 5:**
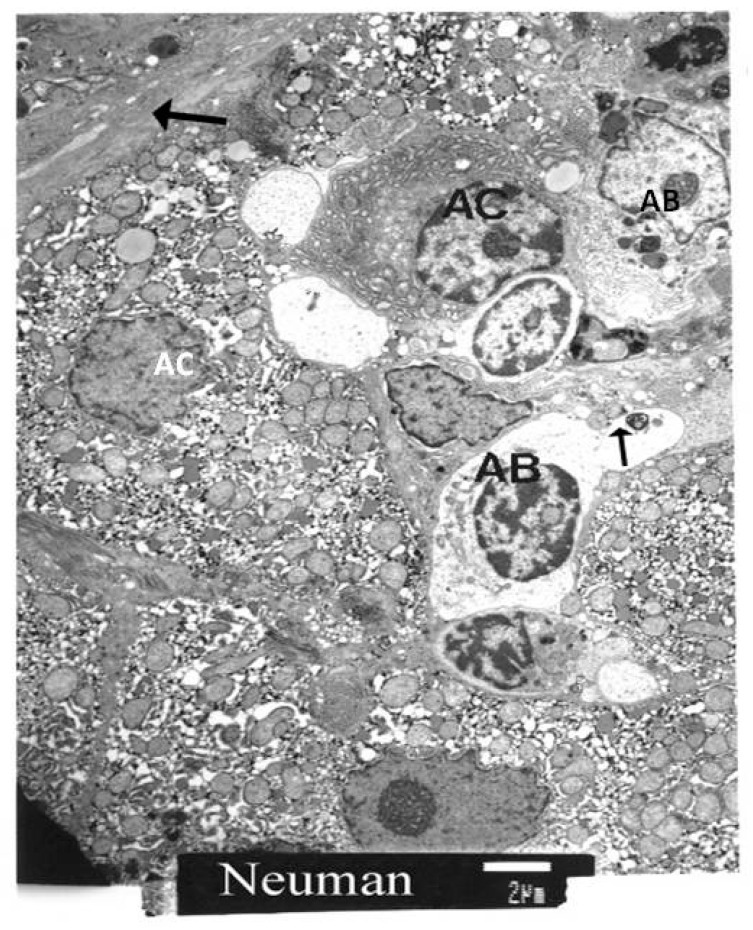
TEM of a biopsy. A shrunken apoptotic cell with a shrunken chromogen nucleus is at the bottom of the micrograph. Other apoptotic cells are scattered in the micrograph. Apoptotic bodies (AB) can be observed. The arrow points to fragments of cellular debris.

In the next micrograph (Figure 5), we present the findings from another biopsy. There are apoptotic bodies (AB) and apoptotic cells (AC). One apoptotic cell contains condensed chromatin. Large deposits of collagen are visible in the left upper corner of the micrograph.

## 5. Discussion

This article emphasizes the increased need to focus on molecular biology markers of inflammation and repair in patients with HCV. Due to our better understanding of the molecular biology of RNA viruses, such as HCV, we have been able to highlight the mechanism of inflammation and repair in HCV.

RNA viruses and protein and nucleic acid products of infection or replication, including single-stranded or double-stranded RNA and polyuridine signatures, have been identified as viral PAMPs. Toll-like receptors (TLRs) or nucleic acid binding proteins serve as PAMP receptors [7,22,23,24,25,26,27,28,29,30].

In the case of HCV, the viral RNA contains each of these PAMP signatures and is sufficient to trigger a host response when introduced into naive cells [31,32]. Our work has demonstrated that the cellular retinoic acid-inducible gene I (RIG-I), a double- stranded RNA, PAMP receptor and transducer of the host response, is critical for HCV RNA PAMP signaling [27]. In hepatocytes, (the target cell of HCV infection), the independent pathways of RIG-I and TLRS signaling comprise two major pathways that trigger the host defense by dsRNA [36].

The major effect of PAMP receptor engagement is the activation of latent cellular transcription factors that mediate the rapid onset of gene expression, thus marking the immediate–early phase of the host response. Interferon regulatory factors (IRFs) [36] are prominently active in this response. IRF-3 and IRF-7 are activated through viral PAMP-responsive signaling cascades that culminate with their nuclear translocation and transcription effector actions. 

In parallel, the transactivation of IRF-1, and direct chromatin remodeling, result in the assembly of a complex with IRF-3 on the IFN-p promoter, leading to a transcriptional response that produces secreted IFN-p from the infected cell [36,37]. The secreted IFNs engage the local tissue through autocrine and paracrine processes of binding the IFN-a/gamma receptors. This results in activation of the Jak-STAT pathway, in which the receptor-associated Jak and Tyk1 protein kinases catalyze the phosphorylation of signal transducer and activator of transcription (STAT) proteins on critical serine and tyrosine residues. This confers STAT activation and stable association with IRF-9. The resulting ISGF3 transcription factor complex localizes to the cell nucleus, where it binds to the IFN-stimulated response element (ISRE) within the promoter/enhancer region of IFN-stimulated genes (ISGs) [36]. Jak-STAT signaling leads to a second wave of transcriptional activity, marking ISG expression in the infected cell. The paracrine effects of IFN-p induce ISG expression within the neighboring uninfected cells of the local tissue to induce an antiviral state that limits cell-to-cell virus spread. Many PAMP receptors and their constituent signaling partners are ISGs, and though expressed basally at a low level that facilitates surveillance, their levels increase following IFN production [37]. In the human liver, this most likely serves to enhance the sensitivity of signaling in infected tissue [38,39,40].

Our studies on inflammation in HCV focused on the immunological responses of the individual. The question is what distinguishes successful natural clearance of the inflammatory process from chronic persistence of the repair process. This personalized knowledge will pave the way for developing precise therapies for patients with HCV. This effort requires continued studies in suitable clinical systems, and in particular cohorts, diverse ethnic populations, varied age groups, and with people who misuse alcohol and drugs. These patients are at risk for developing chronic and severe liver diseases, such as cirrhosis. Cirrhosis is characterized by irregular fibrosis, bile duct multiplication and nodular regeneration of the parenchyma and cytoplasmic, hyalinized, eosinophilic bodies. The levels of TNFα, IL-6 and IL-8 are markedly raised in HCV patients in the present study, and correlate with disease severity [8]. In addition, we show the increased levels of apoptosis and necrosis in HCV-infected individuals. Our studies in HCV patients were based on our experience of studying apoptosis and necrotic markers in liver diseases [8,41,42].

In this study we aimed to measure the circulating levels of IL-10, IL-8 and TNFα in the sera of HCV individuals, and to compare these levels with the same parameters in non-infected, healthy individuals. As shown in Figure 2, the differences between the TNF alpha values in cirrhotic and non-cirrhotic individuals are statistically significant. In addition, the levels of the pro-angiogenic cytokine VEGF is statistically different between cirrhotic and non-cirrhotic HCV-infected patients.

Using these biomarkers in clinical laboratory medicine will enable the clinician to employ a personalized medicine approach to stop inflammation and to induce liver cell repair.

We described the immunoregulatory events, the T helper response profile (Th1, Th2), and the different inflammasome and apoptosome profiles associated with different stages of liver disease severity, which can be observed throughout the evolution of the symptoms.

The present study stimulates high-quality, multi-disciplinary collaborative research. The dysregulation of inflammasomes is associated with cell death. By focusing on a mechanistic approach of understanding inflammasome and apoptosome characteristics in patients with HCV, we may avoid severe liver damage, as well as prevent disease complications.

Understanding the apoptosome and inflammasome in patients with HCV infection permits the laboratory medicine specialist to collaborate with the hepatologist in order to offer precision medicine to their patients. The clinician understands the importance of using the specific biomarkers. The clinician and the laboratory continue to exchange information. The collaboration permits specific personalized therapy for patients. 

## Figures and Tables

**Table 1:** Laboratory parameters in HCV-infected patients.

Laboratory Parameters	Cirrhotic	Non-Cirrhotic	*p*
AST (U/L)	175 ± 62	72 ± 24	<0.05
ALT (U/L)	174 ± 67	54 ± 15	<0.05
GGT (U/L)	202 ± 88	133 ± 12	<0.001
AP (U/L)	195 ± 71	73 ± 21	<0.001
Bilirubin total (mg/dL)	1.76 ± 0.25	1.3 ± 0.4	<0.05

*p* values < 0.05 are considered significant. AST—Aspartate aminotransferase; ALT—Alanine aminotransferase; AP—alkaline phosphatase; GGT—Gamma-Gluthamyl tansferase.

**Table 2:** Cytokines, chemokines, necrosis and apotosis in sera.

Parameter	Normal Values Controls (280) (Mean ± SD)	HCV Patients (88) (Mean ± SD)	*p* Value
IL-1 (pg/mL)	24.0 ± 6.0	85.3 ± 28.0	0.003
IL-6 (pg/mL)	30.0 ± 10.0	65.0 ± 20.0	0.005
IL-8 (pg/mL)	44.0 ± 10.0	182.6 ± 43.0	<0.001
IL-10 (pg/mL)	30.0 ± 5.0	45.0 ± 20.0	NS
IL-12 (pg/mL)	45.0 ± 10.0	58.0 ± 30.0	NS
IL-13 (pg/mL)	12.0 ± 4.0	75.0 ± 10.0	<0.001
IL-17 (pg/mL)	45.0 ± 5.0	98.0 ± 12.0	0.005
TGF beta (ng/mL)	25.0 ± 5.0	88.0 ± 22.0	0.005
M-30 (U/L)	80.0 ± 25.0	159.0 ± 37.0	<0.0001
M-65 (U/L)	120.0 ± 60.0	476.0 ± 118.0	<0.0001

M-30/M-65 (ccK—cleaved caspase cytokeratin (8-M65/18-M30). L—liter; U—units; IL—interleukins; TGFβ—transforming growth factor beta; mL—milliliter; ng—nanogram; pg—picogram.

**Table 3:** Spearman correlations of histology with cell death, TNF-**a**, TGF-b in aera of HCV patients.

Spearman Correlation	M30	M65	TNFα	TGFβ
lobular inflammation 0–3	0.506 *	0.489 *	0.441 *	0.159
ballooning 0–2	0.419 *	0.449 *	0.557 **	0.136
steatohepatitis 0–2	0.340	0.510 *	0.466 *	−0.044

* *p* < 0.05, ** *p* < 0.01.

## Data Availability

The data presented in this study are openly available in each one of the sites. The reported results for clinical evaluation for HCV patients can be found at the Sunnybrook HSC, Toronto, ON, Canada. All the laboratory results for cytokines, chemokines and apoptosis markers, as well as special virology, can be found at In Vitro Drug Safety and Biotechnology, Toronto, Canada.

## Abbreviations

ALT	alanine aminotransferase (glutamic pyruvic transaminase, GPT)
ALP	alkaline phosphatase
AST	aspartate aminotransferase (glutamic oxalic transaminase, GOT)
CCK	Caspase-cleaved cytokeratin (8 and 18) M-30 and M-65
DAMPs	danger-associated molecular patterns
EBV	Epstein–Barr virus
EGF	endothelial growth factor
ELISA	enzyme-linked immunosorbent assay;
FasL/TNFSF6	factor-related apoptosis ligand
Fas	ligand, a member of tumor necrosis factor super-family
FDA	Food and Drug Administration
GGT	γ-glutamyl transferase
HBV	hepatitis virus B
HCC	hepatocellular carcinoma
HCV	hepatitis virus C
HCV-RNA	hepatitis C virus ribonucleic acid
HHV6	human herpes virus 6
IFN	interferon
IL	interleukin
INR	international normalized ratio
IRF	interferon regulatory factor
ISGs	IFN-stimulated genes
ISRE	IFN-stimulated response element
IU/L	international units/liter
Jak	Janus kinase
mean ± st. d	mean ± standard deviation
MIP	macrophage inflammatory protein-1 (CCR5 ligand)
MMP	matrix metalloprotease
NFκB	nuclear factor-κB
NLRP	NOD-like receptor pyrin domain
PAI -1	plasminogen activator inhibitor 1
PAMPs	pathogen-associated molecular patterns
pro-CASP-1	procaspase 1
PICP	C-terminal procollagen I peptide
PIIINP	amino-terminal propeptide of procollagen type III
PRRs	pattern recognition receptors
PYCARD	apoptosis-associated speck-like protein containing a CARD ASC
RANTES (CCL5)	regulated upon activation normal T cell expressed and secreted
RNA	ribonucleic acid
TGF	*β*-transforming growth factor beta
TIMP	tissue inhibitor of matrix metalloproteinase
TNF-α	tumor necrosis factor alpha
TLR	Toll-like receptors
VEGF	vascular endothelial growth factor
